# The level of acute alcohol exposure during binge drinking associates with the extent of cardiac response

**DOI:** 10.1007/s00392-025-02722-4

**Published:** 2025-08-19

**Authors:** Aenne S. von Falkenhausen, Christina Krewitz, Raphaela Winter, Anna Kern, Dorothee Brunner, Stefan Brunner, Moritz F. Sinner

**Affiliations:** 1 Department of Medicine I, LMU University Hospital, LMU Munich, Ziemssenstr. 5, 81377 Munich, Germany; 2 German Centre for Cardiovascular Research (DZHK), partner site: Munich Heart Alliance, Munich, Germany; 3 Department of Anesthesiology, LMU University Hospital, LMU Munich, Munich, Germany; 4 Task Force Infectious Diseases, Bavarian Health and Food Safety Authority, Munich, Germany; 5 Center for Sports Medicine, LMU University Hospital, LMU Munich, Munich, Germany

**Keywords:** Alcohol, Arrhythmia, Electrocardiogram, Holiday heart syndrome, Autonomic nervous system

## Abstract

**Introduction:**

The cardiovascular effects of acute alcohol exposure remain incompletely understood, despite its reported association with arrhythmias like atrial fibrillation (AF). The Munich-BREW II study supported a link between excessive alcohol consumption, elevated heart rate, impaired heart rate variability (HRV), and increased arrhythmia incidence. Here, we present sub-analyses exploring how the amount of congested alcohol during binge drinking and the maximum breath alcohol concentration (BAC) influence these findings.

**Methods:**

The Munich-BREW II study is a prospective, single-center cohort study conducted at LMU University Hospital, Munich between October 2016 and July 2017. Participants consumed alcohol under supervision, with hourly BAC measurements and continuous 3-lead Holter monitoring for ECG analyses of heart rate, HRV, and arrhythmias. Subgroup analyses stratified participants by quartiles of alcohol consumption and peak BAC, respectively.

**Results:**

We analyzed 193 participants (mean age 29.9 ± 10.6 years, 36% women). Subgroup analyses revealed that higher alcohol intake during binge drinking correlated with significantly elevated heart rate (*p* < 0.001) and suppressed HRV measures (SDNN, *p* = 0.003; RMSSD, *p* = 0.001). Similarly, higher BAC levels were associated with increased heart rates (*p* < 0.001) and both reduced SDNN (*p* < 0.001) and RMSSD (*p* = 0.002). Both subgroups indicated a more pronounced effect in the highest quartile. Clinically relevant arrhythmias were not differentially distributed across subgroups.

**Discussion:**

In this subgroup analysis of the Munich-BREW II study, higher alcohol intake and BAC during binge drinking were associated with increased heart rate and suppressed cardiac autonomic tone. The results suggest a dose response relation and discourages excessive alcohol use. Further research will need to investigate the degree of alcohol exposure to modify clinical outcomes.

**Graphical abstract:**

Illustrates key results—**A**: The exemplary glasses are filled with the beverages consumed at the median level by men and women, respectively. The fill level in each glass corresponds to the median amount of beverage consumption in liters. Colors represent types of beverages (yellow: beer, pale orange: liquor, orange: long drinks and red: wine). **B** and **C**: Boxplots represent the number of drinks and the breath alcohol concentration (BAC) for men and women, respectively. The median is illustrated by a black line, the mean by a red dot. Boxes indicate interquartile range. **D**: Graphs visualize the trend of heart frequency (red) and standard deviation of R-R intervals (SDNN; yellow) with increasing breath alcohol concentration (from left to right)

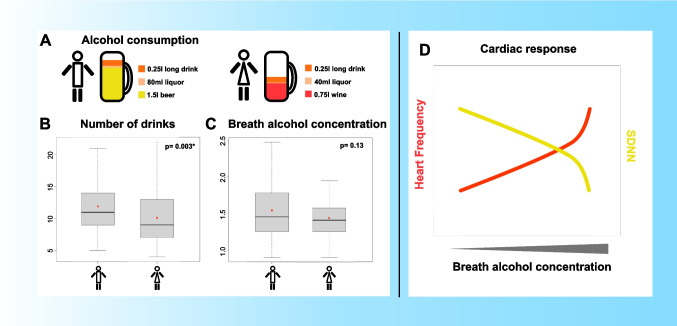

## Introduction

Alcohol consumption is a global health threat. Yet, cardiovascular effects of acute alcohol exposure and especially binge drinking are insufficiently understood. Despite anecdotal reports suggesting reduced cardiovascular mortality with moderate chronic use [1, 2], more important findings highlight the ‘Holiday Heart Syndrome’ of acute alcohol exposure triggering cardiac arrhythmias, especially atrial fibrillation (AF) [3–5].

Recently, the Munich-BREW II study supported that excessive drinking during binge drinking associates with inappropriately high heart rate, interferes with cardiac autonomic tone reflected by heart rate variability (HRV) markers, and results in an excess of clinically relevant arrhythmias [6]. Impaired HRV is further linked to cardiovascular mortality [7–9]. Despite clear overall results, it remains unclear, to what extent the amount of consumed alcohol and the peak breath alcohol concentration (BAC) influence the reported findings. Here, we present respective sub-analyses of the Munich-BREW II study.

## Methods

The Munich-BREW II study is an investigator-initiated, prospective, single arm, single center cohort study conducted at the LMU University Hospital of the Ludwig-Maximilians-Universität (LMU), Munich, Germany. The study and its study protocol were approved by the Ethics Committee at the Ludwig-Maximilians-Universität (accession number 616–16). Study design and overall findings have been reported [6]. Briefly, we prospectively enrolled individuals planning to acutely consume alcohol (binge drinking) between October 2016 and July 2017. All participants provided written informed consent and were ≥ 18 years old. We excluded individuals with a history of AF, with a cardiac implantable device, and pregnant or breastfeeding women.

After enrollment, participants consumed alcohol in public or private locations supervised by the study team. We measured breath alcohol concentration (BAC) at study start and then hourly until the end of the ‘drinking period’, which was defined as the time period in which each participant consumed alcohol. All consumed alcoholic beverages were documented. Beverages were categorized as beer (0.5 l; 20 g ethanol), wine (0.25 l; 20 g ethanol), liquor (0.02 l; 6.2 g ethanol), and long drink (0.04 l of liquor; 12.4 g ethanol).

From the beginning of the drinking period and for overall 48 h, all participants were monitored by 3-lead, cable-free patch Holters (Mini Holter Recorder, Medset-Medizintechnik GmbH, Hamburg, Germany) allowing for a systematic analysis of heart rate and HRV measures including the standard deviation of R-R intervals (SDNN) and the square root of mean squared differences of successive R-R intervals (RMSSD). From the ECG recordings, we extracted the hour of recording during which the maximum BAC was measured. In this period, we averaged heart rate and calculated SDNN and RMSSD. We then stratified our cohort based on the following two criteria for targeted subgroup analyses: (a) quartiles of the absolute alcohol consumption in grams based on the individual alcohol intake during the study period, and (b) quartiles of the maximum documented BAC.

Statistically, we present data as absolute and relative frequencies and compare them using Fisher’s exact tests. Continuous data are presented as means ± standard deviation compared by ANOVA or as medians (25th;75th percentiles) compared by Kruskal-Walli’s tests. P-values < 0.05 are considered significant. Analyses were performed using R (version 4.3.1, The R Foundation for Statistical Computing, Vienna, Austria).

## Results

We analyzed 193 individuals (mean age 29.9 ± 10.6 years, 70 (36%) women) [6]. Men had a significantly higher BMI than women (24.8 ± 3.1 vs. 22.2 ± 3.0, *p* < 0.001). Study participants were thus generally young and healthy adults without a relevant cardiovascular risk profile (see Table 1). Considering alcohol consumption, women preferred wine (3 (0;6) vs. 0 (0;2.5) units, *p* < 0.001), whereas men consumed more beer (3 (2;6) vs. 0 (0;3) units, *p* < 0.001) or liquor (4 (2;6) vs. 2 (0;5) units, *p* < 0.001, Table 1). Overall, men consumed significantly more drinks (11 (9;14) vs. 9 (7;13) units, *p* = 0.003), resulting in a numerically higher amount of alcohol compared to women (142.2 (118.6;183.4) g vs. 132.4 (92.2;184.1) g, *p* = 0.05, Table 1). We observed no difference in the maximum BAC between men and women (1.46 (1.26;1.79) g/kg in men vs. 1.42 (1.26;1.58) g/kg in women, *p* = 0.13) (Graphical abstract).

**Table 1:** Cohort characteristics

Parameter	All *n *= 193	Male *n *= 123	Female *n *= 70	*p*
Age, years	29.9 ± 10.6	29.6 ± 9.8	30.5 ± 11.8	0.55
Body mass index (BMI), (kg/m2)	23.8 ± 3.3	24.8 ± 3.1	22.2 ± 3.0	< 0.001
**Cardiovascular risk profile**				
Arterial hypertension, n (%)	6 (3.1)	3 (2.4)	3 (4.3)	0.67
Diabetes mellitus, n (%)	0 (0)	0 (0)	0 (0)	n.a
Cigarette Smoking, n (%)	29 (15.0)	22 (17.9)	7 (10.0)	0.21
High cholesterol, n (%)	4 (2.1)	1 (0.8)	3 (4.3)	0.14
Family history for cardiovascular disease, n (%)	24 (12.4)	19 (15.5)	5 (7.1)	0.11
**Drinking habits**				
Beer (n)	2 (0;5)	0 (0;3)	3 (2;6)	< 0.001*
Wine (n)	0 (0;4)	3 (0;6)	0 (0;2.5)	< 0.001*
Hard liquor (n)	3 (1;6)	2 (0;5)	4 (2;6)	< 0.001*
Long drink (n)	1 (0;3)	1 (0;3)	1 (0;3.5)	0.55
Alcohol total (g)	140.0 (112.4;183.4)	132.4 (92.2;184.1)	142.2 (118.6;183.4)	0.05
Drinks total (n)	10 (8;14)	9 (7;13)	11 (9;14)	0.003*

Continuous variables are mean ± standard deviation or median [25th;75th percentile]

* indicates statistical significance

We subsequently stratified our cohort as described and compared the indicated ECG measures across (a) quartiles of the absolute alcohol consumption, and (b) quartiles of the maximum documented BAC. Detailed results are presented in Table 2 and visualized in Fig. 1. Participants with higher absolute alcohol consumption (a) exhibited a significantly higher median heart rate across quartiles of absolute alcohol intake (*p* < 0.001). Both SDNN (*p* = 0.003) and RMSSD (*p* = 0.001) were continuously and significantly suppressed during the hour of maximum BAC with an increasing intake of alcohol. Likewise, individuals with higher maximum BAC levels (also analyzed in quartiles) presented with a significantly higher median heart rate (*p* < 0.001) as well as lower SDNN (*p* < 0.001) and RMSSD (*p* = 0.002) values. Importantly, there were no differences in resting heart rate or heart rate variability between participants, regardless of total alcohol consumption or maximum BAC, at 24 h post-peak BAC—a reference timepoint when the acute effects of alcohol would have largely subsided (Table 2). Additionally, participants with higher alcohol consumption (in grams) during study period reported a higher average weekly alcohol consumption while participants who reached higher breath alcohol concentrations (BAC) during the study did not necessarily report significantly greater weekly alcohol intake. As previously reported, clinically relevant arrhythmias occurred in 5.2% of our cohort. Yet, we did not observe differences in the incidence of these arrhythmias, when stratifying our cohort as done here.

**Table 2:** Electrocardiogram findings, stratified by measures of alcohol consumption

Parameter	All *n* = 193	Alcohol consumption (g)				*p*	BAC (g/kg)				*p*
		Q1 ≤ 112.4 *n* = 49	Q2 > 112.4 & ≤ 140.0 *n* = 47	Q3 > 140.0 & ≤ 183.4 *n *= 45	Q4 > 183.4 *n *= 46		Q1 ≤ 1.26 *n *= 50	Q2 > 1.26 & ≤ 1.44 *n *= 49	Q3 > 1.44 & ≤ 1.67 *n *= 46	Q4 > 1.67 *n *= 48	
HR (bpm) without alcohol	71 (60;81)	70 (60;76)	74 (65;82)	68 (59;79)	74 (61;87)	0.13	71 (62;80)	73 (64;79)	71 (60;86)	73 (59;86)	0.97
SDNN (ms) without alcohol	89 (66;119)	93 (75;129)	79 (61;118)	95 (69;112)	88 (66;111)	0.48	91 (56;127)	82 (69;118)	90 (69;115)	96 (65;114)	0.99
RMSSD (ms) without alcohol	41 (27;61)	50 (33;73)	36 (26;48)	43 (30;61)	42 (26;57)	0.14	39 (24;66)	37 (29;59)	44 (29;57)	43 (29;66)	0.91
HR (bpm) in h of max BAC	99 (88;109)	90 (82;99)	104 (91;109)	98 (85;107)	106 (96;117)	< 0.001*	93 (84;104)	95 (85;106)	102 (88;109)	110;99;118)	< 0.001*
SDNN (ms) in h of max BAC	55 (42;72)	66 (55;77)	56 (44;69)	56 (45;72)	48 (35;65)	0.003*	58 (49;74)	61 (48;78)	59 (43;75)	45 (32;55)	< 0.001*
RMSSD (ms) in h of max BAC	20 (15;29)	25 (20;33)	20 (15;27)	21 (15;28)	17 (12;25)	0.001*	22 (15;28)	25 (17;34)	21 (15;30)	18 (12;25)	0.002*
Clinically relevant arrhythmias (n)	10 (5.2%)	3 (6.1%)	3 (6.4%)	1 (2.2%)	3 (6.5%)	0.79	4 (8.0%)	4 (8.2%)	2 (4.3%)	0 (0%)	0.18
Bradycardic (n)	6 (3.1%)	3 (6.1%)	1 (2.1%)	1 (2.2%)	1 (2.2%)	0.73	2 (4.0%)	2 (4.1%)	2 (4.3%)	0 (0%)	0.58
Tachycardic (n)	4 (2.1%)	0 (0%)	2 (4.3%)	0 (0%)	2 (4.3%)	0.26	2 (4.0%)	2 (4.1%)	0 (0%)	0 (0%)	0.34
Chronic alcohol consumption per week (g)	271 ± 295	152 ± 182	249 ± 303	253 ± 262	378 ± 324	< 0.001*	230 ± 268	268 ± 321	221 ± 253	363 ± 316	0.07

*BAC* Blood alcohol concentration; *HR* heart rate; *SDNN* standard deviation of R-R intervals; *RMSSD* square root of mean squared differences of successive R-R intervals; “without alcohol” refers to the time 24 h after the maximum BAC level as reference period

* indicates statistical significance

## Discussion

We present subgroup analyses of the Munich-Brew II study stratifying by the amount of consumed alcohol and by the maximum BAC. Participants with higher absolute alcohol consumption showed significantly higher cardiac excitation as indicated by higher median heart rate, and showed modulated cardiac autonomic tone as indicated by significantly lower SDNN and RMSSD values. Whereas similar in both subgroups, the effect was more pronounced in those reaching higher BAC compared to those who merely consumed more absolute alcohol in grams. Our data thus suggest that it is primarily the acute degree of drunkenness, measured by BAC, that mirrors the acute cardiac effects of alcohol consumption. The individual BAC level needs to be reached individually, and the amount of alcohol required to reach this BAC depends on many factors including sex and BMI. Other factors, unmeasured in our current study, may further include the level of physical fitness or individual blood pressure, liver function, and concomitant food intake [10–12] as well as how blood alcohol levels correlated to BAC levels.

Our findings align with prior research demonstrating dose-dependent effects of alcohol on cardiac electrophysiology and arrhythmogenic risk. Notably, two large-scale meta-analyses showed a clear association between increasing alcohol consumption and heightened risk of atrial fibrillation [13, 14]. These studies support the notion that even moderate alcohol intake can influence cardiac rhythm, and that the risk escalates with higher doses. While our study focuses on acute effects during binge drinking, the observed increases in heart rate and reductions in heart rate variability may represent early autonomic changes that precede or contribute to arrhythmic events, especially under repeated exposure. By demonstrating that maximum BAC has a stronger correlation with cardiac parameters than absolute alcohol intake alone, our results offer a mechanistic link to the dose–response relationship observed in chronic alcohol exposure studies. These insights underscore the importance of considering both the quantity and the intensity of alcohol exposure when assessing cardiovascular risk.

In conclusion, our analysis again highlights the importance of recognizing that acute alcohol exposure during binge drinking, and particularly the maximally reached BAC, is associated with negative cardiac effects, namely an inappropriately increased heart rate and modulated cardiac autonomic tone. Although possibly due to sample size limitations and hence not found in our subgroups, but as reported before, such acute alcohol exposure has also been linked to the occurrence of clinically relevant arrhythmias [6]. The autonomic consequences of acute alcohol exposure during binge drinking have further been linked to adverse cardiac outcomes and increased cardiac mortality [7–9]. We thus emphasize the importance of further studies to substantiate the association between acute alcohol consumption and arrhythmias most importantly in more vulnerable patients with known cardiac diseases. Even more we encourage comprehensive risk education to reduce excessive alcohol use and binge drinking in the first place.
